# Complete Genome and Calcium Carbonate Precipitation of Alkaliphilic *Bacillus* sp. AK13 for Self-Healing Concrete

**DOI:** 10.4014/jmb.1908.08044

**Published:** 2019-11-01

**Authors:** Yoonhee Jung, Wonjae Kim, Wook Kim, Woojun Park

**Affiliations:** 1 Laboratory of Plant Breeding and Seed Technology, Department of Biosystems and Biotechnology, Korea University, Seoul, 0284, Republic of Korea; 2 Laboratory of Molecular Environmental Microbiology, Department of Environmental Sciences and Ecological Engineering, Korea; 3 University, Seoul 02841, Republic of Korea

**Keywords:** Bacillus species, microbially-induced calcium carbonate precipitation, calcium acetate, isotope-ratio mass spectrometry, X-ray diffraction, crack healing

## Abstract

Bacteria that are resistant to high temperatures and alkaline environments are essential for the biological repair of damaged concrete. Alkaliphilic and halotolerant *Bacillus* sp. AK13 was isolated from the rhizosphere of *Miscanthus sacchariflorus*. Unlike other tested *Bacillus* species, the AK13 strain grows at pH 13 and withstands 11% (w/v) NaCl. Growth of the AK13 strain at elevated pH without urea promoted calcium carbonate (CaCO_3_) formation. Irregular vateritelike CaCO_3_ minerals that were tightly attached to cells were observed using field-emission scanning electron microscopy. Energy-dispersive X-ray spectrometry, confocal laser scanning microscopy, and X-ray diffraction analyses confirmed the presence of CaCO_3_ around the cell. Isotope ration mass spectrometry analysis confirmed that the majority of CO_3_^2-^ ions in the CaCO_3_ were produced by cellular respiration rather than being derived from atmospheric carbon dioxide. The minerals produced from calcium acetate-added growth medium formed smaller crystals than those formed in calcium lactate-added medium. Strain AK13 appears to heal cracks on mortar specimens when applied as a pelletized spore powder. Alkaliphilic *Bacillus* sp. AK13 is a promising candidate for self-healing agents in concrete.

## Introduction

Biogeochemical cycles are affected by microbial processes, including mineral precipitation, which changes the Earth’s surface [1-3]. Microbially-induced calcium carbonate precipitation (MICP) commonly occurs in environments such as soil, cave, freshwater, and marine environments [4-7]. In the process of calcium carbonate precipitation, four key environmental factors control MICP-chemical reactions: calcium concentration, dissolved inorganic carbon (DIC) concentration, pH, and nucleation site availability [3]. Different microorganisms need different compositions of MICP parameters to promote MICP [8]. Bacteria such as *Bacillus*, *Lysinibacillus*, and *Sporosarcina* species appear to produce calcium carbonate crystals under suitable conditions [9]. Microbial metabolic processes such as ureolysis, photosynthesis, denitrification, ammonification, sulfate reduction, and methane oxidation induce calcium carbonate precipitation due to elevated pH during bacterial metabolism [10-15]. The role of bacterial surfaces on calcium carbonate precipitation is controversial. Boundeleva *et al*. reported the failure of finding calcium carbonate on or near live cells [16]. A recent study by Zhang *et al*. concluded that negatively charged cell surfaces do not act as nucleation sites for calcium carbonate [17]. However, calcium carbonate formation on bacterial cell surfaces has been well-documented in other reports [18]. Bacterial surfaces having extracellular polymeric substances (EPS) are believed to promote MICP by providing nucleation sites to lower energy barriers [19, 20].

Until now, MICP has been widely used in various fields: biotechnology, geotechnology, paleobiology, and civil engineering [21]. In environmental engineering, MICP is used in bioremediation to remove heavy metals from contaminated environments. MICP can also alter soil properties by strengthening soil texture [22-25]. Self-healing concrete that repairs micro-cracks by MICP is an active area of research in civil engineering. Cracks occur in concrete structures for several reasons, including autogenous shrinkage, freeze-thaw reactions, and compressive and tensile forces [25]. These sporadic defects decrease the durability of concrete structures, incurring high repair costs; healing such cracks with calcium carbonate precipitation autonomously and immediately after they appear is an economical solution [25]. Unlike chemical methods in which concrete repair is initiated by injecting self-healing agents into cracks, using bacterial metabolism to produce self-healing via calcium carbonate precipitation is considered an eco-friendly alternative [3]. However, conditions during and after concrete production are inhospitable to bacterial survival [26-28]. Conditions inside concrete are harsh because of lack of nourishment, high alkalinity, and high salt concentrations [27]. The ability of calcium carbonate-precipitating bacteria to endure those conditions is crucial to perform as self-healing agents. However, most studies in this field have not examined whether the bacteria could survive in severe structural environments such as concrete. One of the most common bacteria used for biotechnology, especially in the bio-concrete field, are species belonging to the genus *Bacillus*. This Gram-positive bacterium is a rod-shaped aerobic or facultative anaerobic prokaryotic cell that is 1–10 μm long [29]. One reason that the *Bacillus* species is preferred for MICP processes is that they can simultaneously increase the pH of the neighboring environment and produce CO_2_ by decomposing urea and by cellular respiration, creating efficient conditions for MICP [6, 30]. *Bacillus* species can also form spores that are useful for the treatment of construction surface cracks. They also work in mortar, which presents harsh conditions for bacterial survival [31-33]. However, urea decomposition can produce a large amount of unwanted by-product: ammonia. This by-product negatively affects both the environment and human health [34]. In addition, ammonia production causes reinforcement corrosion, which negatively affects the concrete structure [35]. Ammonia accumulation also inhibits the MICP process, resulting in less than optimal biomineralization [36]. Inducing MICP without urea by utilizing *Bacillus* species should be a priority in biotechnological innovation for self-healing concrete. Therefore, non-ureolytic MICP using alkaliphilic *Bacillus* sp. AK13 could be applied to harsh concrete environments. The results of this study suggest that the source of calcium, initial pH, and moderate sugar content were linked to the growth of AK13 cells. Different calcium sources altered the morphology of minerals and amount of biofilm. Furthermore, our data indicate that many CO_3_^2-^ ions are produced via cellular respiration.

## Material and Methods

### Whole Genome Sequencing and Genome Comparison Analysis

Genomic DNA of *Bacillus* sp. AK13 was extracted using the Wizard Genomic DNA purification kit (Promega, USA). Whole-genome sequencing was performed using PacBio RSII Single-Molecule Real-Time (SMRT) sequencing (Pacific Biosciences, USA) with a SMRTbell template library from Chunlab (Korea), according to the manufacturer’s instructions. The complete genome was achieved using the CLgenomics program provided by Chunlab. Gene annotation and assembly were done using the NCBI Prokaryotic Genomes Automatic Annotation Pipeline. The CLgenomics program provided by Chunlab, KEGG pathway searching, and GView server were used for genome comparison between AK13 and other alkaliphilic *Bacillus* species. Proteins from each *Bacillus* species adjacent to strain AK13 and the neutrophile *Bacillus subtilis* were compared using OrthoVenn. The complete genome sequence of strain AK13 can be found in GenBank (Accession number: CP042163). The 16S rRNA sequence of AK13 also can be found in GenBank (Accession number: MK517564).

### Examination of MICP

Colonies isolated from the stock plate were incubated overnight in Luria-Bertani (LB) broth at 37°C, and 10^6^ CFU/ml of overnight culture in LB broth was inoculated into a 125 ml flask containing B4 medium composed of 0.25% calcium acetate, 0.4% yeast extract, and 0.5% glucose or modified B4 medium (B4L) composed of 0.25% calcium lactate, 0.4% yeast extract, and 0.5% glucose. Both media were adjusted to approximately pH 11.2 (range 11.1–11.4) with filtered 3M NaOH. Growth, pH change, and calcium utilization for evaluating non-ureolytic MICP were monitored in two growth media. Growth and pH change were also tested in LB medium at pH 11. Cultures were incubated at 37°C for 5 to 9 days to observe bacterial MICP. Aerobic conditions were created using vigorous agitation at 220 rpm. The growth rate of strain AK13 was determined by counting the colony-forming units (CFU) with three growth media. In addition, pH changes and the rate of calcium ion utilization were monitored periodically using a pH electrode and a calcium-ion selective electrode (ISE) (Orion Versa Star Advanced Electrochemistry Meter, Thermo Fisher Scientific, USA). This experiment was conducted in triplicate. Cells were cultured in B4 and B4L medium for 5 to 9 days, and the induced spores were analyzed using the CFU assay after boiling at 80°C for 10 min to detect spore formation by strain AK13.

### Field-Emission Scanning Electron Microscopy (FE-SEM), Energy-Dispersive X-Ray Spectrometry (EDS), and X-Ray Diffraction (XRD) Analyses of MICP

AK13 was incubated in B4 medium and B4L medium before FE-SEM and EDS analyses. The cells were fixed with low-strength Karnovsky’s solution (2% paraformaldehyde, 2.5% glutaraldehyde, and 0.1 M phosphate buffer at pH 7.2) for 2 h and fixed with 2%osmium tetroxide solution for 2 h. The fixed samples were gradually dehydrated with ethanol (30%, 50%, 70%, and 100%) for 10 min at each step and placed onto an aluminum stub for 4 days to dry. The samples were coated with platinum and analyzed using a Quanta 250 FEG field-emission scanning electron microscope (FEI; Thermo Fisher Scientific) and EDS mapping. The lyophilized powder of precipitated calcium carbonate-cell composites from MICP observed in B4 medium cultures was examined using XRD for mineral identification with Dmax2500/PC (Japan).

### Confocal Laser Scanning Microscopy (CLSM) Analysis of Biofilm and Calcium Carbonate Development

Biofilms from AK13 were stained for 30 min with FilmTracer SYPRO Ruby biofilm matrix dye at room temperature and visualized using CLSM (LSM800; Carl Zeiss, Germany). FilmTracer SYPRO Ruby biofilm matrix-stained biofilm cells were used to obtain confocal images under red fluorescent light (excitation wavelength: 450 nm, emission wavelength: 610 nm). To visualize calcium carbonate, calcein (Sigma) was added to the B4 medium and incubated for seven days so that the CaCO_3_ formation environment was constructed, and confocal images under green fluorescent light with a reflection signal of excitation between 483 and 493 nm were obtained. Biofilms were evaluated for the density of morphology, and precipitated calcium carbonate was evaluated for morphology, height, and density (C-Apochromat 40x/1.20 W Korr M27; Carl Zeiss).

### Isotope-Ratio Mass Spectrometry (IRMS) Analysis

Media containing different amounts of stable isotope-labeled glucose were used to trace the origin of CO_3_^2-^ ions. Four samples with 0%, 20%, 60%, and 100% D-glucose ^13^C_6_ in pH 11 B4 medium containing 0.5% glucose were prepared. After seven days of incubation at 37°C, the minerals from each sample were harvested and tested for the presence of the heavier carbon isotopes using IRMS (Delta V advantage).

### Examination of Alkalophilicity and Halotolerance

Growth under alkaline and saline conditions was measured at various pH values (pH 6–13) and NaCl concentrations (0%–11%) in LB broth at ambient temperature using a microplate absorbance reader (Tecan). An aliquot containing 10^6^ CFU/ml of strain AK13 was washed twice with phosphate-buffered saline (PBS) and inoculated into 48-well microtiter plates containing B4L medium. Microtiter plates were incubated at 37°C for 72 h in static conditions.

### Cultivation of *Bacillus* sp. AK13 for Spray Drying

*Bacillus* sp. AK13 were cultured in modified sporulation medium ‘A’ for three days in a 1 L flask. The components of the medium were agricultural by-products, such as oil cake. AK13 spore powders were made by using the SD-Basic model spray dryer (LabPlant, UK). Spray drying conditions were 165°C inlet temperature, 1.9 MPa airflow volume, and level 5 pump speed (45% full speed, 100 ml/6 min). 10 g of spore powder was harvested when 1 L of medium A was spray dried.

### Pelletizing Spore Powder and Producing Mortar Specimens

To pelletize the cellulose-based spore powder, yeast extract, calcium lactate, and diatomite were mixed, and polyvinyl acetate (PVA) was included as a binding agent. Nutrient availability was confirmed using agitation culture. A portion (1 g) of each pellet was diluted 1:10 (w/v) in distilled water (DW). Bacteria were cultured at 30°C and 220 rpm in a 26 × 150 mm test tube. The 0-day sample was used after a 10 min culture. Ordinary Portland Cement (OPC), standard sand, and 15.3% (w/v) AK13 spore pellets were mixed to produce the mortar specimens (40 × 40 × 10 mm^3^). Control specimens were made by mixing pellets composed of diatomite and PVA.

### *In situ* Survival Test and Crack-Healing Test

Pellet-mixed mortars were frittered and diluted 1:10 (w/v) in PBS. Diluted samples were vortexed for 10 min and incubated for 10 min at 30°C and 220 rpm. They were then centrifuged at 160 rpm for 10 min. The supernatant was harvested and diluted with PBS. Colonies on the pH 8 LB agar plates were counted after 48 h of incubation. Crack healing was observed on the pH 8 LB agar plate after breaking the specimens. To maintain a wet state, 5 ml autoclaved DW was added to the specimens daily using a pipet on a clean bench. The specimens were cured in an incubator at 30°C. Crack widths were measured in triplicate using the UVP ColonyDoc-It Imaging station program (Analytik Jena, Germany).

## Results

### Whole-Genome Sequence and Phylogenetic Tree of Alkaliphilic Bacteria AK13

**Fig. 1:**
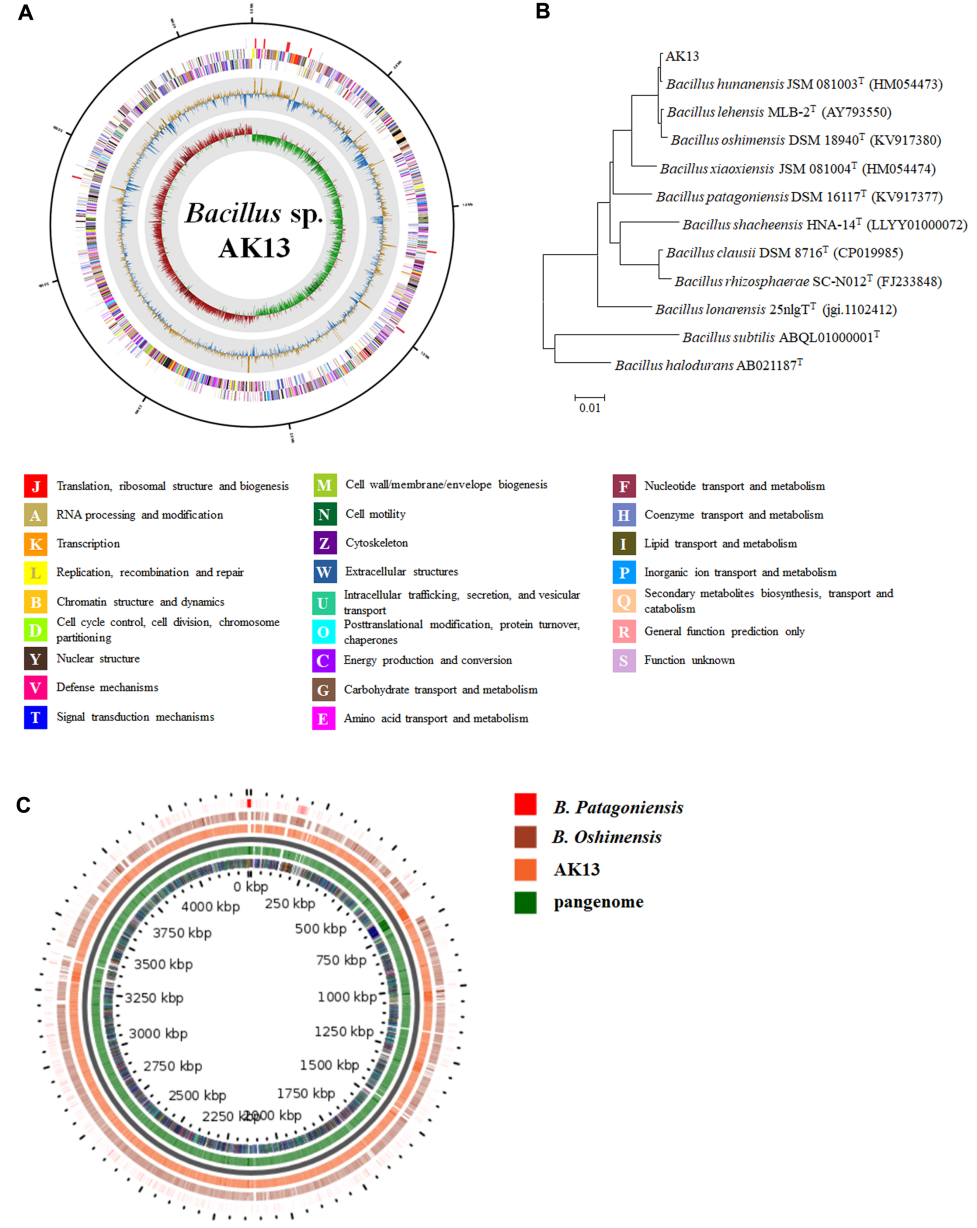
Whole-genome sequencing and genome comparison analysis between AK13 and other alkaliphilic *Bacillus*. (**A**) Circular genomic map of *Bacillus* sp. AK13. From the outside to the center are RNA genes, genes on the reverse strand, genes on the forward strand, GC ratio, and GC skew. Genes are colored according to their clusters of orthologous group (COG) category. (**B**) Phylogenetic neighborjoining tree of strain AK13 (**C**) comparative genomics of strain AK13, *B. oshimensis* DSM 18940 ^T^, and *B. patagoniensis* DSM 16117 ^T^.

We conducted whole-genome sequencing of *Bacillus* sp. AK13 DNA. A complete genome comprising one circular chromosome of 4,250,608 base pairs and a GC content of 40.04% was obtained (Fig. 1A). The phylogenetic tree of strain AK13 was evaluated with one 16S rRNA gene sequence. The strain AK13 was phylogenetically identified as a *Bacillus* species (Fig. 1B). Its full 16S rRNA gene sequence of length 1,548 bp showed high similarity with those of *B. hunanensis* JSM 081003 ^T^ (99.86%), *B. lehensis* MLB-2^T^ (99.73%), and *B. oshinensis* DSM 18940 ^T^ (99.52%). These 16S rRNA gene sequences also shared similarities with those of *B. xiaoxiensis* JSM 081004 ^T^ (98.07%) and *B. patagoniensis* DSM 16117 ^T^ (96.94%). These bacterial strains all have alkaliphilic or alkali-tolerant features. Since genomic sequences were only available for *B. oshinensis* DSM 18940 ^T^ and *B. patagoniensis* DSM 16117 ^T^, these two species were used for the analysis. The highest average nucleotide identity (ANI) value was 91.05% when strain AK13 was compared with *B. oshinensis* DSM 18940 ^T^. In pangenome comparisons of two other *Bacillus* species, strain AK13 had a backbone similar to that of *B. oshinensis* but not *B. patagoniensis* (Fig. 1C). The genome size of AK13 was 4.25 Mb, which is intermediate between those of the two other *Bacillus* species. *Bacillus* sp. AK13 had the highest number of tRNA and rRNA genes among the three *Bacillus* species. Because the alkaliphilic *Bacillus* species uses sodium ions and protons to control pH homeostasis, the number of coding sequences (CDSs) related to inorganic ion transport and metabolism (P) and energy production and conversion (**C**) was determined. The number of CDSs in the amino acid transport and metabolism (**E**) clusters of orthologous group (COG) group was also determined because genes in this group produce ammonia to create alkaline conditions. The number of CDSs related to the P COG group in strain AK13 was the smallest among the three alkaliphilic *Bacillus* species, and the number of CDSs in the C group was intermediate (Table S1). The number of CDSs in the E COG group in AK13 was also intermediate among the three *Bacillus*, but within the AK13 strain, the E COG group had the largest number of CDSs among all COG groups. To confirm the genes that are essential for the survival of the alkaliphilic *Bacillus* strains, the strain AK13 and 12 alkaliphilic or alkali-tolerant *Bacillus* that are adjacent to AK13 and one non-alkaliphilic *Bacillus subtilis* str. 168^T^ were compared. The alkaliphilic *Bacillus* species were *B. oshimensis* DSM 18940^T^, *B. patagoniensis* DSM 16117^T^, *B. clausii* DSM 8716^T^, *B. lonarensis* 25nlg^T^, *B. shacheensis* HNA-14^T^, *B. rhizosphaerae* SC-N012^T^, *B. pseudalcaliphilus* DSM 8725^T^, *B. hemicellulosilyticus* JCM 9152^T^, *B. plakortidis* P203^T^, *B. alkalinitrilicus* DSM 22532^T^, *B. okhensis* Kh10-101^T^, and *B. ligniniphilus* L1^T^. Four proteins are common to all 13 alkaliphilic species and absent from the *B. subtilis* str. 168^T^ species (Table S2). The proteins are DEAD/DEAH box helicase, an Rne/Rng family ribonuclease, an HU family DNA-binding protein, and an aldehyde dehydrogenase family protein. These four proteins are involved in alkaliphilic *Bacillus* environmental adaptation.

### Alkaliphilic and Halotolerance Properties of *Bacillus* Strain AK13

**Fig. 2:**
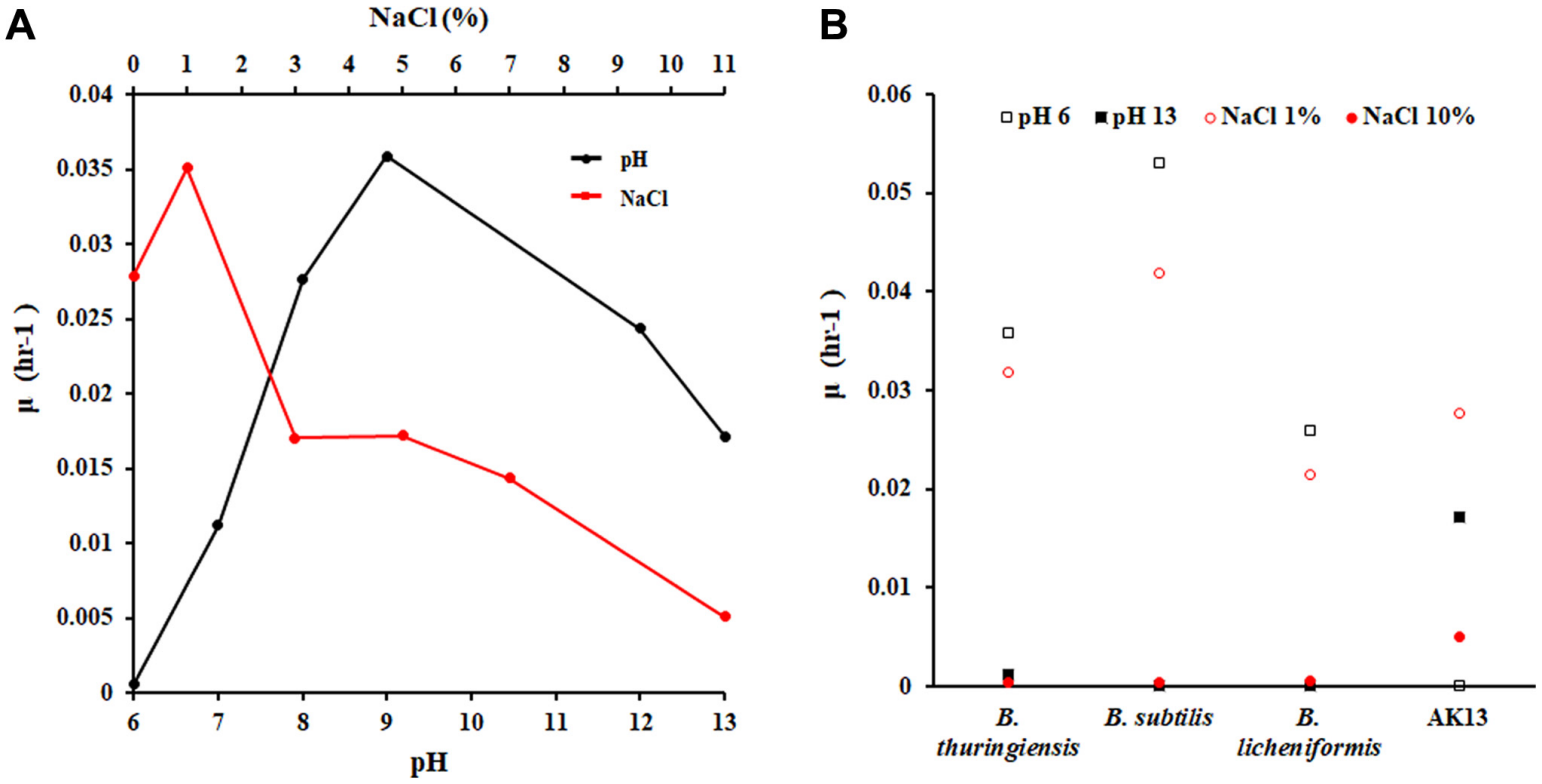
Alkaliphilic and halotolerance properties of *Bacillus* genre AK13. (**A**) Growth in alkaline and high salt conditions. The specific growth rate (μ) is the average of three determinations of growth rate during the exponential phase. (**B**) Growth of *B*. sp. AK13 and other *Bacillus*, including *B. thuringiensis*, *B. subtilis*, and *B. licheniformis* in different LB medium conditions.

Calcium carbonate-precipitating bacteria should be able to endure harsh conditions such as undernourishment, alkalinity, high temperature, and high salinity during and after the concrete manufacturing process [26-28]. *Bacillus* strains are known to tolerate alkaline conditions [27], and strain AK13 showed alkalophilicity. Strain AK13 grows in media with a pH of up to 13 and in salt (NaCl) concentrations above 11% (Fig. 2A). In extremely high pH and salt concentration media, the lag phase was longer, and the growth rate was slower. Strain AK13 did not grow in pH 6 medium, and its growth was better in 1% NaCl medium than in 0% NaCl medium. Strain AK13 was better able to adapt to high alkaline and salinity environments compared with other *Bacillus* bacteria (Fig. 2B). Strain AK13 grew under alkaline conditions of pH 13 and high salt conditions, but other tested *Bacillus* species did not grow under such conditions.

### pH Alteration and Calcium Utilization during Biomineralization

**Fig. 3:**
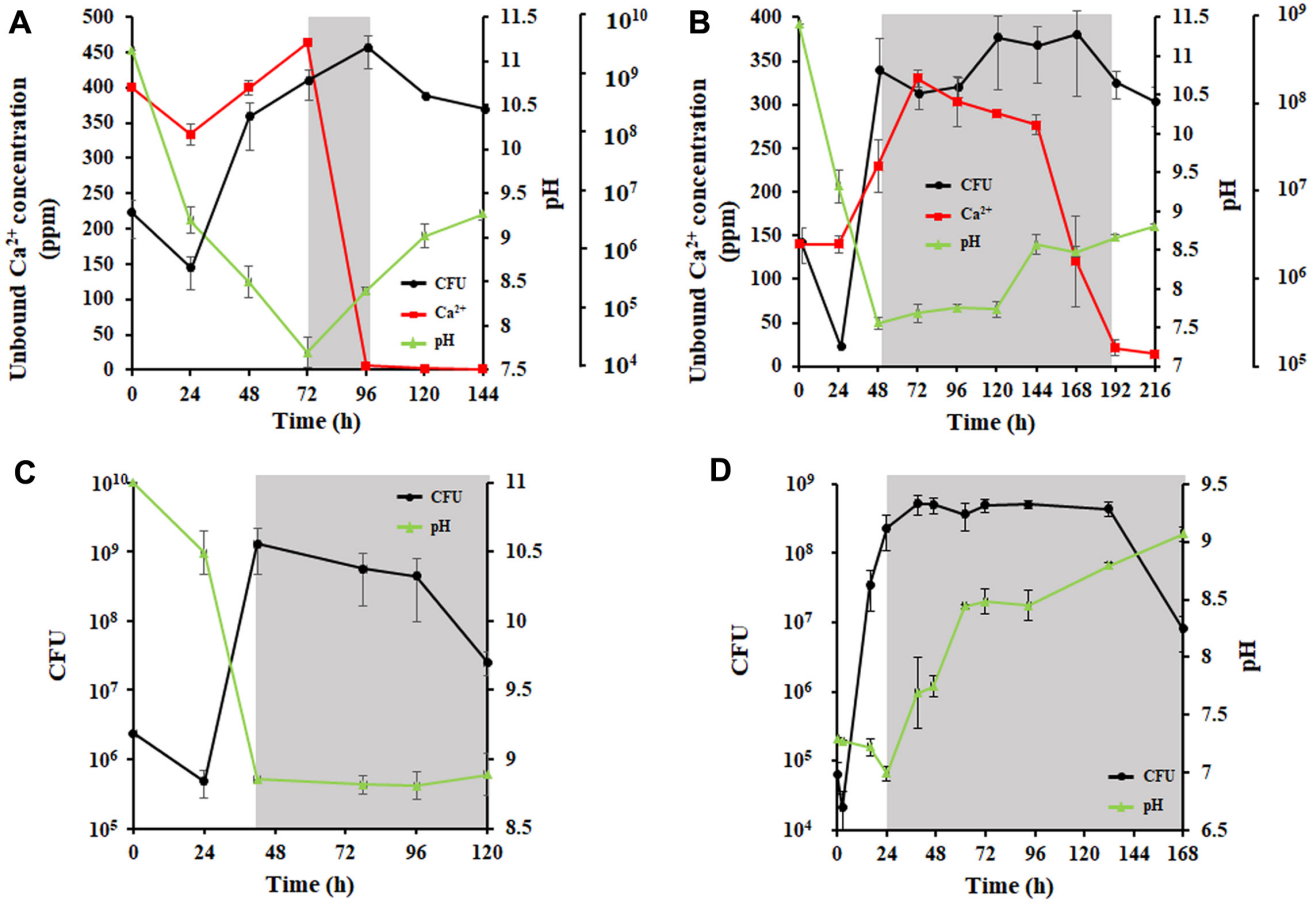
Monitoring of growth, pH changes, and calcium utilization in B4 medium, B4L medium with calcium lactate, LB medium with pH adjustment to approximately 11.2, and LB medium with pH adjustment to 7.3. (**A**) pH 11.1 B4 medium; (**B**) pH 11.4 B4L medium; (**C**) pH 11 LB medium; and (**D**) pH 7.3 LB medium.

Because MICP formation is related to pH, free calcium ions, and bacteria, changes in CFU, pH, and unbound calcium ion concentrations over time were analyzed. Three media—B4, B4L, and LB—were used to measure the effect of different nutrients on AK13 growth and MICP. Because strain AK13 is alkaliphilic and considering the effect of this strain on concrete conditions, the initial pH of the media was adjusted to approximately 11.2 (pH range 11.0–11.4). All three factors had close relationships with one another. In B4 medium, the pH initially dropped to 7.7 during the exponential stage (Fig. 3A). In this stage, the unbound calcium ion concentration rose likely due to the dissociation of ions from the bacterial surface and the pH decrease [37]. As the strain AK13 entered the stationary growth phase between 72 h and 96 h and the growth rate decreased, the pH started to rise, and the unbound calcium ion concentration began to decline. After this time, the ion concentration fell sharply within 24 hours. After 96 h, the concentration of the unbound calcium ion decreased slightly to less than 10 ppm. The pH of the medium increased to 9.2 after 144 h of incubation, and the maximum cell density of strain AK13 was 2.14 × 10^9^ (± 1.7 × 10^9^) CFU/ml.

In B4L medium, which contains calcium lactate instead of calcium acetate, three factors—CFU, pH, and free calcium ion—were also correlated but had slightly different results than those in B4 medium (Fig. 3B). The initial pH decreased, and the concentration of the unbound calcium ion increased in the early growth stage as they did in B4 medium. However, the pH did not rebound immediately after reaching stationary phase, nor did the calcium ion concentration immediately decrease. These two factors took longer to change in B4L medium than in B4 medium, but the time needed to reach the stationary phase decreased to 48 h. Moreover, the pattern of calcium ion change was different. The decrease in unbound calcium ion concentration was more gradual than in B4 medium; in B4 medium, the decrease was rapid over a shorter time. The final pH was approximately 8.8, and the maximum cell concentration in B4L medium was 4.8 × 10^8^ (± 3.8 × 10^8^) CFU/ml, which is slightly lower than is observed in B4 medium. The calcium ion consumption in B4 medium was more efficient than in B4L medium, and virtually all the calcium ions were consumed in a shorter time. To confirm the growth of AK13 in medium without calcium ions, growth and pH change were analyzed in LB medium (Fig. 3C). The time to reach the maximum cell concentration was the shortest in LB medium, and the pH decreased before the stationary phase as it did in the two other media, but the degree of decrease was less in LB medium. After the cells reached the stationary phase, the pH stayed constant at approximately 8.8 until the end of the experiment, a value that is similar to that reached in the B4L medium. When the initial pH of LB medium was adjusted into pH 7.3, the pH rose with same trend to the stationary phase and the final pH was approximately 9 (Fig. 3D). To characterize the relationship of initial pH and glucose content to growth, changes in three factors were tested in B4, B4L, and LB media with different initial pH values and glucose contents. In B4 and B4L media with an initial of pH 10, the CFU of strain AK13 increased until 96 h, but sharply decreased after that time (Figs. S1A and S1B). The concentration of unbound calcium ions and pH fluctuated as they did in the experiment with pH 11.2 media, but these factors remained at the same level as the CFU sharply decreased. However, the growth of strain AK13 in pH 10 media was different when the medium composition changed. By reducing the amount of glucose from 0.5% to 0.2% in both media, the three factors behaved as they did in pH 11.2 media. However, the transition time points for both pH and calcium ion content occurred earlier in both media (Figs. S1C and S1D). This result suggests that initial pH and glucose content affect the growth of strain AK13.

### Non-Ureolytic MICP of Strain AK13 in B4 Medium and B4L Medium

**Fig. 4:**
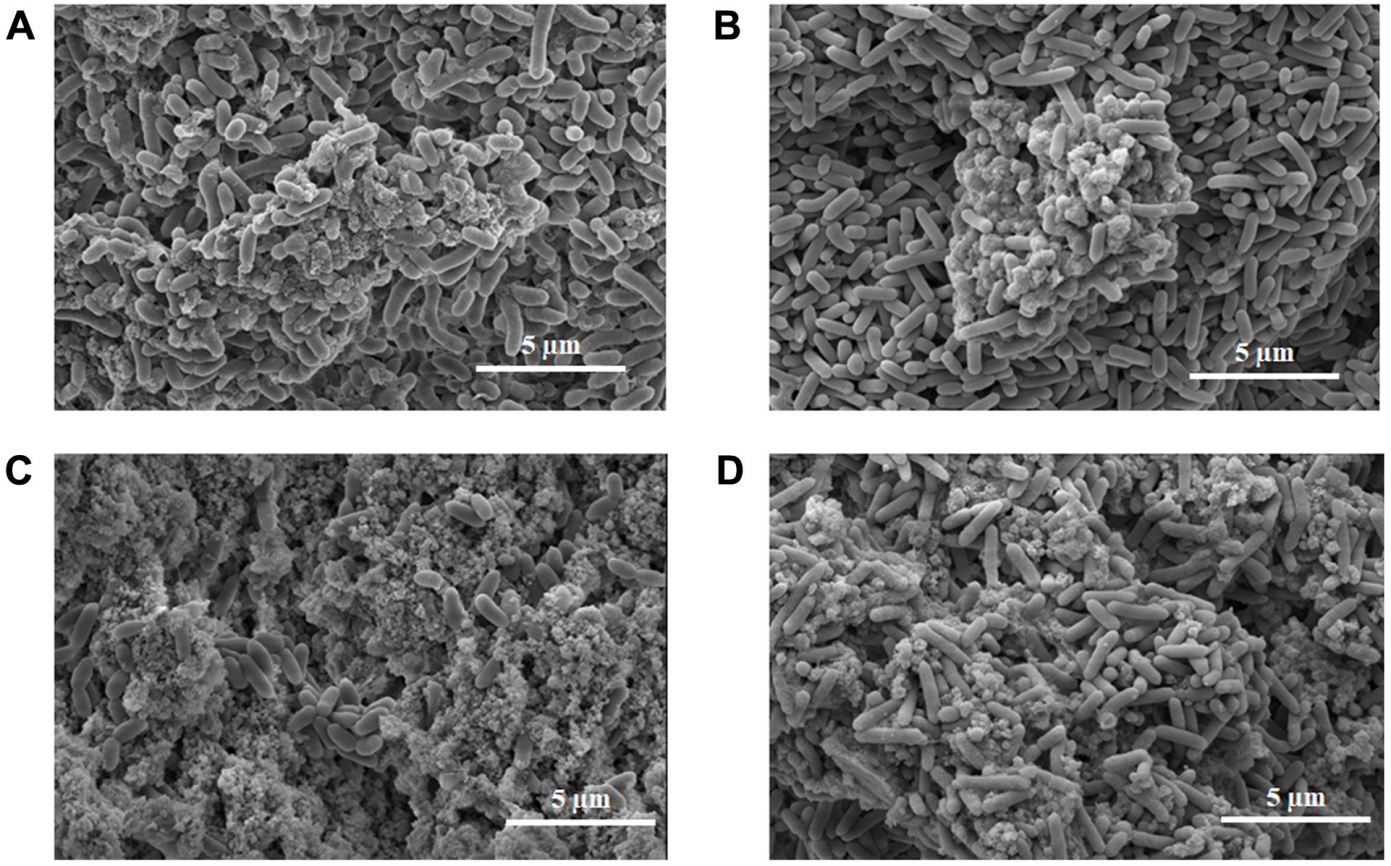
Field-emission scanning electron microscopy (FE-SEM) of strain AK13 in B4 medium and B4L medium. (**A**) Microbially-induced calcium carbonate precipitation (MICP) of strain AK13 in B4 medium after three days of incubation. (**B**) MICP of strain AK13 in B4L medium after seven days of incubation. (**C**) MICP of strain AK13 in B4 medium after 4 days of incubation. (**D**) MICP of strain AK13 in B4L medium after 8 days of incubation. All images are shown at10,000 × magnification.

**Fig. 5:**
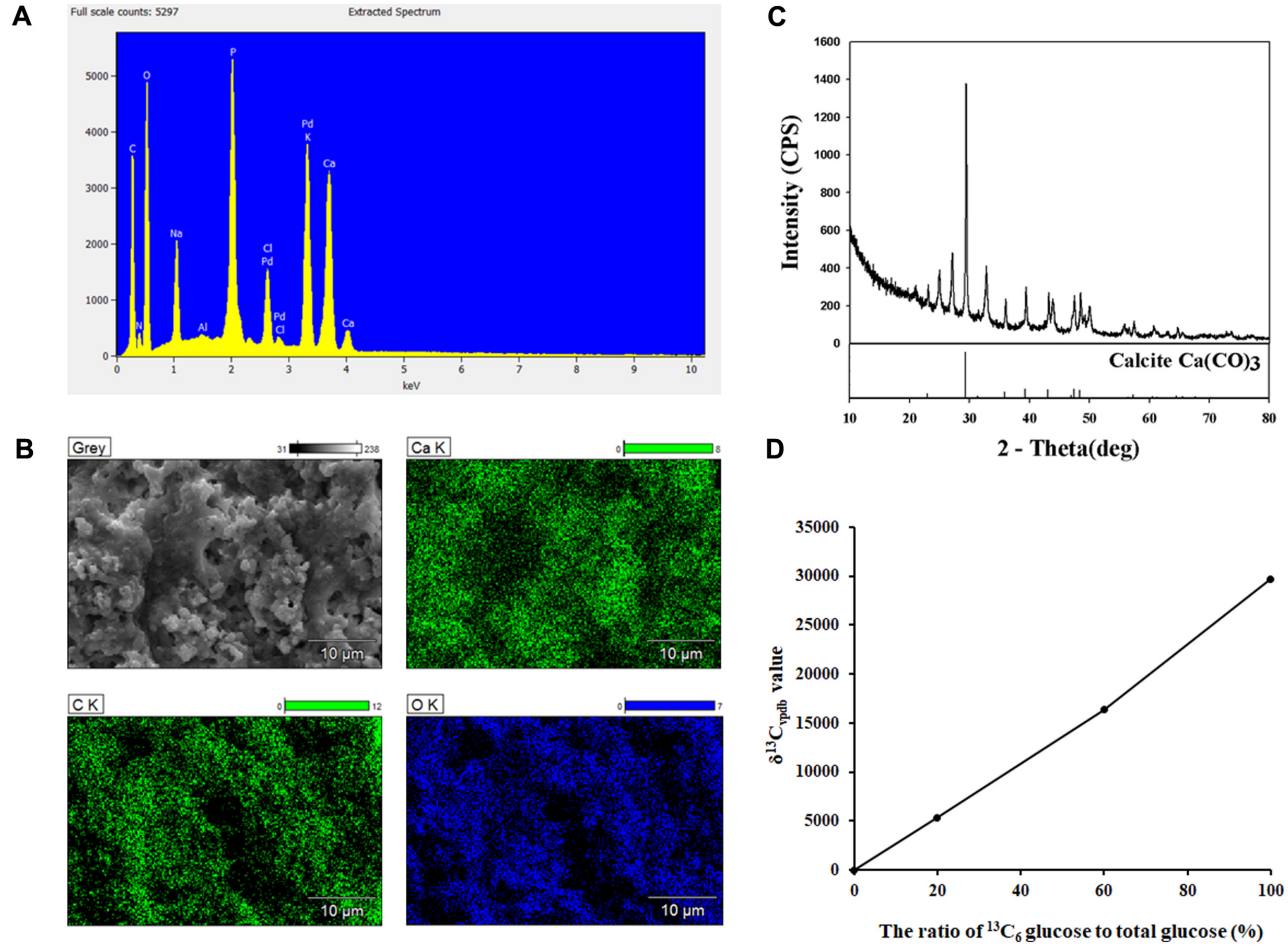
Energy-dispersive X-ray spectrometry (EDS) and X-ray diffraction (XRD) of strain AK13 in B4 medium with non-ureolytic biomineralization conditions. Isotope ratio mass spectrometer (IRMS) analysis of samples with different ^13^C_6_ glucose ratios. (**A**) Calcium peaks indicate the presence of calcium in a mineral form as a result of microbially-induced calcium carbonate precipitation (MICP) by strain AK13. The x-axis is ion-specific voltage signals (keV) and the y-axis is relative proportion of x-ray (x-ray intensity). (**B**) Calcium ion and carbon and oxygen elements that match the mineral distribution in the SEM image indicate the presence of MICP by strain AK13. (**C**) Several peaks verify the formation of calcium carbonate. (**D**) The δ^13^C_vpdb_ value increases in proportion to the ratio.

**Fig. 6:**
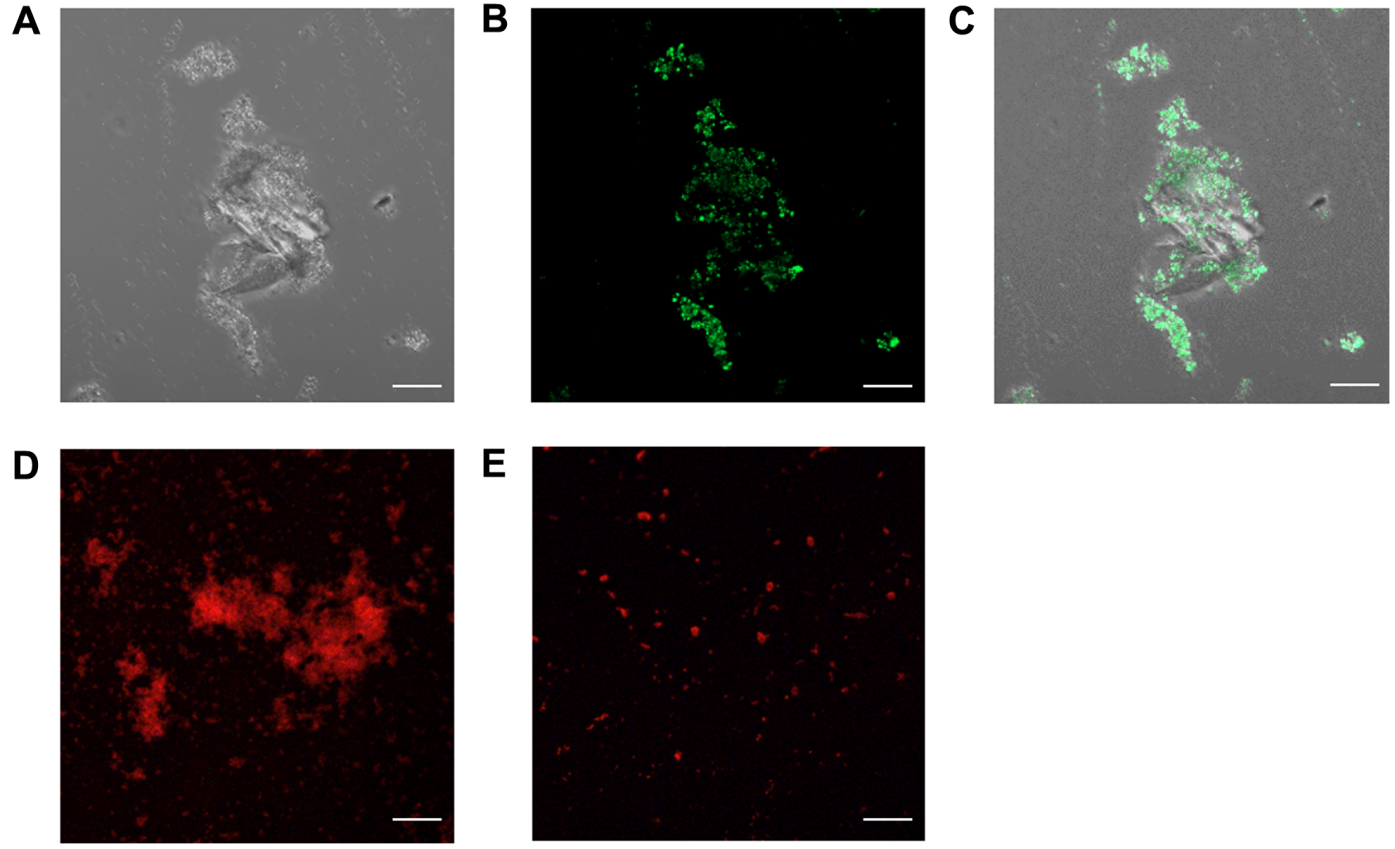
Confocal laser scanning microscopy (CLSM) image of calcium carbonate and biofilm produced by AK13 and observation of crack healing after seven days of incubation. (**A**) DIC image, (**B**) calcein staining image, and (**C**) merge image of CaCO_3_ precipitation site with calcein staining. Scale bars indicate 20 μm. (**D**) Biofilm in B4 medium that contains calcium acetate. (**E**) Biofilm in B4L medium that contains calcium lactate instead of calcium acetate.

To observe the mineral pattern induced by strain AK13, the calcium carbonate precipitation over time in B4 and B4L medium was analyzed using FE-SEM. After 72 h of inoculation, when the pH of the B4 medium reached the point of transition to a pH increase, calcium carbonate precipitates started to form (Fig. 4A). After 96 h, many calcium carbonate crystals formed, resulting in a dramatic decrease in unbound calcium ion concentration. In B4L medium, crystal precipitate formation began at approximately 168 h (Fig. 4B). Moreover, the mineral morphology differed between the two media. Compared to minerals formed in B4L medium, the MICP in B4 medium exhibited smaller morphology (Figs. 4C and 4D). The minerals induced by strain AK13 were attached to individual cells as mineral-bacteria clusters. Minerals that precipitated in B4 medium were verified as calcium carbonates using EDS analysis, indicating the presence of calcium, carbon, and oxygen (Fig. 5A). The result of EDS mapping also indicated the formation of calcium carbonate. The positions of the calcium ions and the carbon and oxygen elements were directly matched with the precipitation (Fig. 5B), which was confirmed to be calcium carbonate by XRD analysis (Fig. 5C). Minerals precipitated in B4 medium were also visualized by CLSM. When calcein was added to samples that had incubated for seven days under conditions suitable for CaCO_3_ formation, the minerals appeared as green-colored clusters with irregular forms (Figs. 6A-6C). Therefore, strain AK13 induced calcium carbonate precipitation without urea hydrolysis.

Because the contributing source of the CO_3_^2-^ ion for CaCO_3_ precipitation remained unclear, media with different ratios of stable isotope-labeled glucose were used to discover the source of the CO_3_^2-^ ions. After a 7-day incubation at 37°C, the minerals from each sample were harvested and tested for the presence of heavier carbon isotopes using IRMS. The results for the control sample without ^13^C_6_ D-glucose had a slight amount of ^13^C (δ^13^C_vpdb_ + 3.053‰). In comparison, the minerals supplied with ^13^C_6_ D-glucose were highly enriched for the heavier isotope. In a sample with 20% ^13^C_6_ glucose to total glucose, the result was measured at δ^13^C_vpdb_ + 5372.150‰. A sample with 60% ^13^C_6_ glucose was measured at δ^13^C_vpdb_ + 16 332.679‰, and sample with 100% ^13^C_6_ glucose was measured at δ^13^C_vpdb_ + 29 704.694‰. As the ^13^C_6_ glucose ratio was increased by 1, 3, and 5 times, the δ^13^C_vpdb_ value also increased in proportion to the amount of ^13^C (Fig. 5D).

### Biofilm Formation by Strain AK13

Since bacteria can serve as biomineralization nuclei, the ability to attach to bacteria and bacterial cell surface properties are important in bio-concrete. Therefore, biofilm formation was tested strain AK13 by calcium ion presence, which is a key factor in calcium carbonate formation. Biofilm formation increased relative to the calcium contents, suggesting an alteration of surface composition due to calcium ions, a result that agrees with previous findings [33]. CLSM was used to visualize biofilms formed in B4 medium and B4L medium (with calcium lactate instead of calcium acetate) to investigate the effect of calcium ions on biofilm formation. Biofilms formed in calcium lactate medium showed a scattered distribution compared with those formed in calcium acetate medium (Figs. 6D and 6E).

### Nutrient Availability in the Bacterial Pellet, Survivability *in situ*, and Micro-Crack Healing Effect of Spores of *Bacillus* sp. Strain AK13 Mixed in a Mortar

**Fig. 7:**
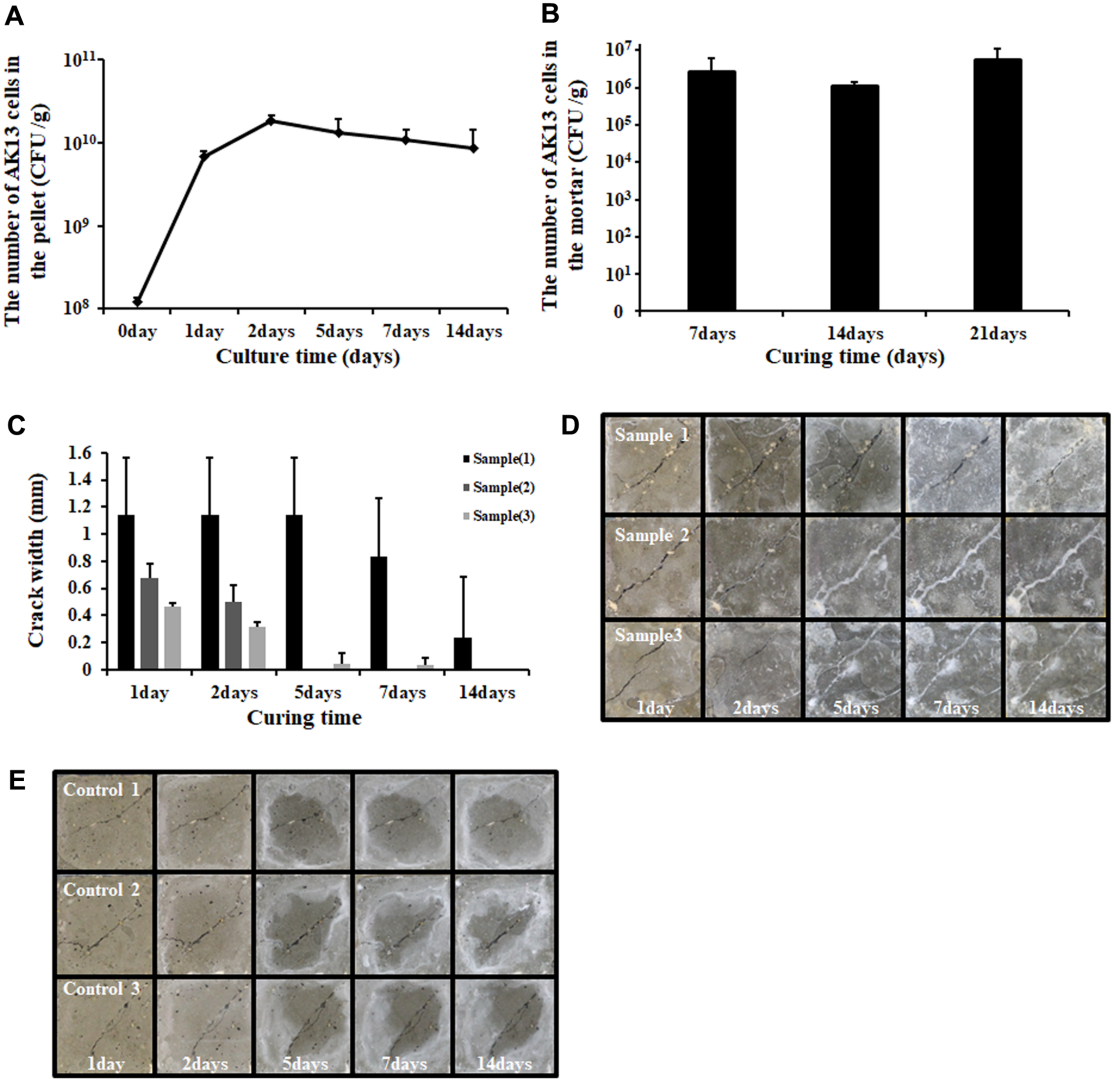
(**A**) Growth curve of pellet diluted 1:10 in distilled water (DW). (**B**) *In situ* survival test for curing time. (**C**) Measurement of crack width on S1, S2, and S3. (**D**) Observation of crack healing on AK13 spore-mixed concretes. (**E**) Observation of crack healing in control specimens.

The number of bacteria in the zero-day pellet was 1 × 10^8^ CFU/g and increased to 1 × 10^10^ CFU/g after one week (Fig. 7A). After two weeks, the number of bacteria remained at 8 × 10^9^ CFU/g. The number of germinated bacteria was 2× 10^6^ CFU/g after 7 days of producing mortar specimens. In 14- and 21-day specimens, the number of bacteria was 1×10^6^ CFU/g and 5 × 10^6^ CFU/g, respectively. The nutrients were sufficient for bacteria and spores to germinate in situ. Five cracks on arbitrary points were selected in Specimens 1, 2, and 3 (S1, S2, and S3). The crack points were observed and measured. Each specimen had a different crack width ranging from 0.45 to 1.70 mm. S1 showed the widest crack with an average width of 1.13 ± 0.42 mm and the slowest healing rate. S2 showed an intermediate crack with an average width of 0.67 ± 0.10 mm and was filled within five days. S3 showed the narrowest crack with an average width of 0.46 ± 0.02 mm, which was filled within ten days. Cracks with an average width of 1 mm were filled within two weeks.

## Discussion

MICP can be induced during the growth and development of microorganisms by alterations in the environment that create conditions suitable for precipitation [3]. Conditions such as high DIC and calcium ion concentration, nucleation site presence, and primarily alkaline pH can be created using the microorganismal metabolism processes of ureolysis, denitrification, and ammonification [10, 13, 14]. In addition to these conditions, the ability to survive in harsh environments such as high salinity or alkaline conditions is crucial for bacteria used in self-healing concrete. Owing to its efficiency, most studies on MICP and its application in self-healing concrete have been performed using the genus *Bacillus*, based on the mechanism of urea hydrolyzation [6]. However, the *Bacillus* genus is not noted for its ability to adapt to the environment inside concrete. In addition, urea decomposition can produce ammonia, an undesired by-product that is harmful to the environment and humans and affects the durability of concrete buildings [35]. Therefore, the aim of this study was to induce calcium carbonate precipitation using alkaliphilic and halotolerant *Bacillus* without urea by other processes.

In this study, *Bacillus* sp. AK13 was successfully applied in self-healing concrete. Complete genome analysis for strain AK13 was performed. This species was classified as new using average nucleotide identity (ANI) analysis. Strain AK13 regulates homeostasis in alkaline and saline conditions using sodium and proton ion transporters [38]. Four proteins were shared by all 13 alkaliphilic species and were absent in *B. subtilis* str. 168^T^ based on comparison with the other 12 alkaliphilic or alkali-tolerant *Bacillus* that are adjacent to AK13 and the non-alkaliphilic B. subtilis (Table S2). The four proteins included DEAD/DEAH box helicase, an Rne/Rng family ribonuclease, an HU family DNA-binding protein, and an aldehyde dehydrogenase family protein. DEAD/DEAH box helicase has been reported to be involved in ribosome assembly and to interact with a membrane-bound degradosome responsible for RNA turnover [39]. The Rne/Rng family of ribonucleases are common proteins that play an essential role in processing 9S rRNA, tRNAs, and the M1 precursor of RNase P transcripts and degrading mRNAs [40]. The DEAD/DEAH box helicase and the Rne/Rng family of ribonucleases may regulate the steady-state concentration of any given RNA as an immediate response of alkaliphilic *Bacillus* to an alkaline environment. The DEAD/DEAH box helicase is reportedly involved in several stress tolerance responses to heat, pH, ethanol, and oxidative stress in bacteria [39]. The HU family of DNA-binding proteins, which is involved in DNA stabilization, may prevent DNA denaturation in an alkaline environment. In addition, the aldehyde dehydrogenase can control pH via production of hydrogen ions. This protein is also involved in glycolysis via restoring NAD^+^.

Many studies on MICP in self-healing concrete have not concentrated on bacterial survival in the unsuitable environments of building structures. *Bacillus* sp. AK13 grows in environments having pH values of up to 13 and salt concentrations above 11%. Strain AK13 may grow better in 1% salt medium than 0% salt medium because alkaliphilic *Bacillus* use sodium ions for pH homeostasis [38]. Therefore, this strain may survive in the cell or spore state when applied inside a concrete and produce calcium carbonate for subsequent crack repair. Besides performing ureolysis, AK13 could increase media pH after it reached the stationary phase, a condition that is conducive to biomineral formation. In all media conditions tested, strain AK13 cultures reached steady state pH levels of approximately pH 9. This characteristic was most clearly observed with LB media with initial pH values of 11 or 7.3 (Figs. 3C and 3D). The initial pH decreases seen with pH 7.3 LB medium may be due to a scarcity of sugar in the yeast extract. The interaction between calcium ions and the bacterial cells during the exponential growth phase has already been studied, and it involves binding of positively charged calcium ions on net negatively charged cell walls before being steadily released [41, 42]. Likewise, the initial concentrations of free calcium ions gradually increased as the growth AK13 proceeded through the exponential phase and sharply decreased after the cultures reached the stationary phase in B4 medium (Fig. 3A). The initial calcium ion increase was higher than that in a previous study using YS11 [37] because the initial pH of the medium was higher, and the pH initially decreased due to the formation of acidic substances like acetate after the consumption of preferred sources such as glucose. *Bacillus* can lower the pH of the medium by utilizing sources like glucose [43]. The degree of pH reduction can vary in different media, but this reduction can inhibit the growth of alkaliphilic AK13 due to changing pH conditions depending on the initial pH and sugar concentration (Fig. S1). Therefore, the proper amount of glucose is crucial for the growth of the bacteria even when the preferred source of strain AK13 is glucose. The decline in calcium ion concentration and increase in pH progressed more slowly in B4L medium than in B4 medium (Fig. 3B). This may be due to efficiency differences between using acetate and lactate. There are more genes in the metabolic pathway of acetate in the AK13 genome than in that of lactate.

The calcium source is a key factor that affects calcium carbonate morphology [44]. The biomineral morphology differed between two culture media tested in this study. The calcium carbonate crystals formed in B4 medium (calcium acetate) showed smaller than those formed in B4L medium (calcium lactate). This suggests that the minerals could develop into different morphologies based on operating conditions [45]. Moreover, the microorganisms in the center of the minerals exerted cellular nucleation effects [20]. Although bacteria have been assumed to be a source of CO_3_^2-^ ions, this had not been confirmed. A previous study investigated the source of CO_3_^2-^ ions by using the stable ^13^C isotope of atmospheric carbon dioxide [4] and confirmed that some of the CO_3_^2-^ ions were derived from atmospheric carbon dioxide. In this study, media containing different proportions of the stable ^13^C isotope of glucose were tested to detect cell-derived CO_3_^2-^ ions. The δ^13^C_vpdb_ value increased in proportion to the amount of ^13^C_6_ glucose. The δ^13^C_vpdb_ value in this study was even higher than that in the atmospheric carbon dioxide results, demonstrating that many CO_3_^2-^ ions are derived from cellular respiration, but the exact proportion of CO_3_^2-^ ions contributed by cellular respiration remains unclear. This result is encouraging because there is insufficient inflow of external carbon dioxide to form minerals inside concrete. The results from biofilm formation demonstrated the role of calcium ion in the formation. It had been suggested that calcium ions might alter bacterial surface components and the composition of EPS-mediated biomineral in biofilm formation [37].

Applying strain AK13 to mortar revealed its crack healing ability. AK13 spores could germinate and grow in concrete with sufficient nutrients. Pellets could construct a suitable environment for AK13 when they encountered water and became untangled. Other sources of water like rain, tap water, and freshwater could contain additional nutrients and be more effective. Moreover, food storage in pellets applied in construction would likely be more efficient than the test results because general construction conditions do not involve agitation as was used in test conditions. Although crack healing was observed using calcium carbonate precipitation, there were some limitations. To create calcium carbonate, the surface of the crack needed to be aerated. Therefore, the filling of cracks on S3, which were smaller than those on S2, was slower because of capillary action. The specimens also need to be optimized via strength and permeability tests.

In conclusion, alkaliphilic and halotolerant *Bacillus* sp. AK13, which forms spores and can precipitate calcium carbonate in the absence of urea, could fill the crack even when applied to mortar. Since the bacteria need simultaneous contact with water and air, unlike other self-healing materials, further investigation on the effect of concrete structural environments—including water flows, rainfalls, and wind gusts—is needed. In addition, further studies on the strength and permeability of the standard specimen are also needed. However, the strain AK13 has potential to act as a crack-healing agent.

## Supplemental Materials



Supplementary data for this paper are available on-line only at http://jmb.or.kr.

## References

[1] Falkowski PG, Fenchel T, Delong EF (2008). The microbial engines that drive Earth's biogeochemical cycles. Science.

[2] Lee YS, Park W (2019). Enhanced calcium carbonate-biofilm complex formation by alkali-generating *Lysinibacillus boronitolerans* YS11 and alkaliphilic *Bacillus* sp. AK13. AMB Express..

[3] De Muynck W, De Belie N, Verstraete W (2010). Microbial carbonate precipitation in construction materials: a review. Ecol. Eng..

[4] Banks ED, Taylor NM, Gulley J, Lubbers BR, Giarrizzo JG, Bullen HA (2010). Bacterial calcium carbonate precipitation in cave environments: a function of calcium homeostasis. Geomicrobiol. J..

[5] Gat D, Ronen Z, Tsesarsky M (2016). Soil bacteria population dynamics following stimulation for ureolytic microbial-induced CaCO_3_ precipitation. Environ. Sci. Technol..

[6] Zhu T, Dittrich M (2016). Carbonate precipitation through microbial activities in natural environment, and their potential in biotechnology: a review. Front. Bioeng. Biotechnol..

[7] Ramos-Cormenzana A, del Moral A, Rivadeneyra MA, Ferrer MR, Delgado R (1994). Precipitation of calcium carbonate by *Vibrio* spp. from an inland saltern. FEMS Microbiol. Ecol..

[8] Hammes F, Verstraete W (2002). Key roles of pH and calcium metabolism in microbial carbonate precipitation. Rev. Environ. Sci. Bio..

[9] Rodriguez-Navarro C, Rodriguez-Gallego M, Chekroun KB, Gonzalez-Munoz MT (2003). Conservation of ornamental stone by *Myxococcus xanthus*-induced carbonate biomineralization. Appl. Environ. Microbiol..

[10] Castanier S, Le Métayer-Levrel G, Perthuisot JP (1999). Ca-carbonates precipitation and limestone genesis - the microbiogeologist point of view. Sediment. Geol..

[11] Dittrich M, Kurz P, Wehrli B (2004). The role of autotrophic picocyanobacteria in calcite precipitation in an oligotrophic lake. Geomicrobiol. J..

[12] Dupraz C, Reid RP, Braissant O, Decho AW, Norman RS, Visscher PT (2009). Processes of carbonate precipitation in modern microbial mats. Earth-Sci. Rev..

[13] Hamdan N, Kavazanjian E, Rittmann BE, Karatas I (2017). Carbonate mineral precipitation for soil improvement through microbial denitrification. Geomicrobiol. J..

[14] Jeong JH, Jo YS, Park CS, Kang CH, So JS (2017). Biocementation of concrete pavements using microbially induced calcite precipitation. J. Microbiol. Biotechnol..

[15] Park SJ, Park JM, Kim W, Ghim SY (2012). Application of *Bacillus subtilis* 168 as a multifunctional agent for improvement of the durability of cement mortar. J. Microbiol. Biotechnol..

[16] Bundeleva IA, Shirokova LS, Bénézeth P, Pokrovsky OS, Kompantseva EI, Balor S (2012). Calcium carbonate precipitation by anoxygenic phototrophic bacteria. Chem. Geol..

[17] Zhang W, Ju Y, Zong Y, Qi H, Zhao K (2018). In situ real-time study on dynamics of microbially induced calcium carbonate precipitation at a single-cell level. Environ. Sci. Technol..

[18] Ghosh T, Bhaduri S, Montemagno C, Kumar A (2019). Sporosarcina pasteurii can form nanoscale calcium carbonate crystals on cell surface. PLoS One.

[19] Kim HJ, Shin B, Lee YS, Park W (2017). Modulation of calcium carbonate precipitation by exopolysaccharide in *Bacillus* sp. JH7. Appl. Microbiol. Biotechnol..

[20] Tourney J, Ngwenya BT (2009). Bacterial extracellular polymeric substances (EPS) mediate CaCO_3_ morphology and polymorphism. Chem. Geol..

[21] Reddy MS (2013). Biomineralization of calcium carbonates and their engineered applications: a review. Front. Microbiol..

[22] Chaturvedi S, Chandra R, Rai V (2006). Isolation and characterization of *Phragmites australis* (L.) rhizosphere bacteria from contaminated site for bioremediation of colored distillery effluent. Ecol. Eng..

[23] Simon MA, Bonner JS, Page CA, Townsend RT, Mueller DC, Fuller CB (2004). Evaluation of two commercial bioaugmentation products for enhanced removal of petroleum from a wetland. Ecol. Eng..

[24] Al-Thawadi SM (2011). Ureolytic bacteria and calcium carbonate formation as a mechanism of strength enhancement of sand. J. Adv. Sci. Eng. Res..

[25] Jonkers HM, Schlangen E Self-healing of cracked concrete: a bacterial approach.

[26] De Belie N, Wang J, Bundur ZB, Paine K, Pacheco-Torgal F, Melchers R, De Belie N, Shi X, Van Tittelboom K, Saez Perez A (2017). Bacteria based concrete. Eco-efficient Repair and Rehabilitation of Concrete Infrastructures.

[27] Gupta S, Dai Pang S, Kua HW (2017). Autonomous healing in concrete by bio-based healing agents-A review. Constr. Build. Mater..

[28] Wang J, Ersan YC, Boon N, De Belie N (2016). Application of microorganisms in concrete: a promising sustainable strategy to improve concrete durability. Appl. Microbiol. Biotechnol..

[29] Wong LS (2015). Microbial cementation of ureolytic bacteria from the genus *Bacillus*: a review of the bacterial application on cement-based materials for cleaner production. J. Clean Prod..

[30] Kim HJ, Eom HJ, Park C, Jung J, Shin B, Kim W (2016). Calcium carbonate precipitation by *Bacillus* and *Sporosarcina* strains isolated from concrete and analysis of the bacterial community of concrete. J. Microbiol. Biotechnol..

[31] Gordon R, Haynes W, Pang C, Smith N (1973). The genus *Bacillus*. Agriculture handbook.

[32] Jonkers HM, Thijssen A, Muyzer G, Copuroglu O, Schlangen E (2010). Application of bacteria as self-healing agent for the development of sustainable concrete. Ecol. Eng..

[33] Lee YS, Park W (2018). Current challenges and future directions for bacterial self-healing concrete. Appl. Microbiol. Biotechnol..

[34] Xu J, Yao W, Jiang Z (2013). Non-ureolytic bacterial carbonate precipitation as a surface treatment strategy on cementitious materials. J. Mater. Civil Eng..

[35] Neville AM (2010). Properties of concrete.

[36] Ow MC, Perwez T, Kushner SR (2003). RNase G of *Escherichia coli* exhibits only limited functional overlap with its essential homologue, RNase E. Mol. Microbiol..

[37] Chaurasia L, Bisht V, Singh L, Singh LP, Gupta S (2019). A novel approach of biomineralization for improving micro and macro-properties of concrete. Constr. Build. Mater..

[38] Lee YS, Kim HJ, Park W (2017). Non-ureolytic calcium carbonate precipitation by *Lysinibacillus* sp. YS11 isolated from the rhizosphere of *Miscanthus sacchariflorus*. J. Microbiol..

[39] Krulwich TA, Ito M, Guffanti AA (2001). The Na+-dependence of alkaliphily in *Bacillus*. BBA-Bioenergetics.

[40] Markkula A, Lindström M, Johansson P, Björkroth J, Korkeala H (2012). Roles of four putative DEAD-box RNA helicase genes in growth of *Listeria* monocytogenes EGD-e under heat, pH, osmotic, ethanol, and oxidative stress conditions. Appl. Environ. Microbiol..

[41] OW MC, Perwez, T, Kushner SR (2003). RNase G of *Escherichia coli* exhibits only limited functional overlap with its essential homologue, RNase E. Mol. Microbiol..

[42] Mishra V (2015). Modelling of the batch biosorption system: study on exchange of protons with cell wall-bound mineral ions. Environ. Technol..

[43] Thomas KJ, Rice CV (2014). Revised model of calcium and magnesium binding to the bacterial cell wall. Biometals.

[44] Pepper RE, Costilow RN (1964). Glucose catabolism by *Bacillus popilliae* and *Bacillus lentimorbus*. J. Bacteriol..

[45] Zhang Y, Guo H, Cheng X (2014). Influences of calcium sources on microbially induced carbonate precipitation in porous media. Mater. Res. Innov..

