# Supplementary material for: Complete Genome and Calcium Carbonate Precipitation of Alkaliphilic Bacillus sp. AK13 for Self-Healing Concrete
Source: J Microbiol Biotechnol. 2019 Nov 1;30(3):404–16. doi: 10.4014/jmb.1908.08044 (PMC9728366; doi:10.4014/jmb.1908.08044)
Supplement: Supplementary file 1 [file JMB-30-3-404-supple.pdf]

J. Microbiol. Biotechnol.
https://doi.org/10.4014/jmb.1908.08044

jmb

Supplement Tables

Table S1. Genomic features of *Bacillus* species AK13 compared with those of other *Bacillus* species including *B. oshimensis* DSM 18940 ^{T} and *B. patagoniensis* DSM 16117 ^{T}

Features	Values		
	AK13	DSM18940^{T}	DSM16117^{T}
Genome size (Mb)	4.25	4.06	4.46
GC content (%)	40.04	39.8	39.6
Contigs	1	1	7
CDS	4,341	4,070	4,709
rRNAs	25	21	22
tRNAs	77	72	74
C COG group	161	156	194
P COG group	225	235	250
E COG group	320	306	326

Table S2. Common proteins in *Bacillus* species AK13 compared with those in 12 other adjacent alkaliphilic *Bacillus* species except for non-alkaliphilic *B. subtilis*

**Common protein**	**Coordinates in AK13**	**Strand**	**Gene name**
DEAD/DEAH box helicase	2,440,459-2,442,075	-	*deaD/cshA*
Rne/Rng family ribonuclease	2,816,072-2,817,556	-	*rng/cafA*
HU family DNA-binding protein	3,058,331-3,058,603	+	
aldehyde dehydrogenase family protein	28,219-29,655	+	*gabD*

Supplement Figure legend

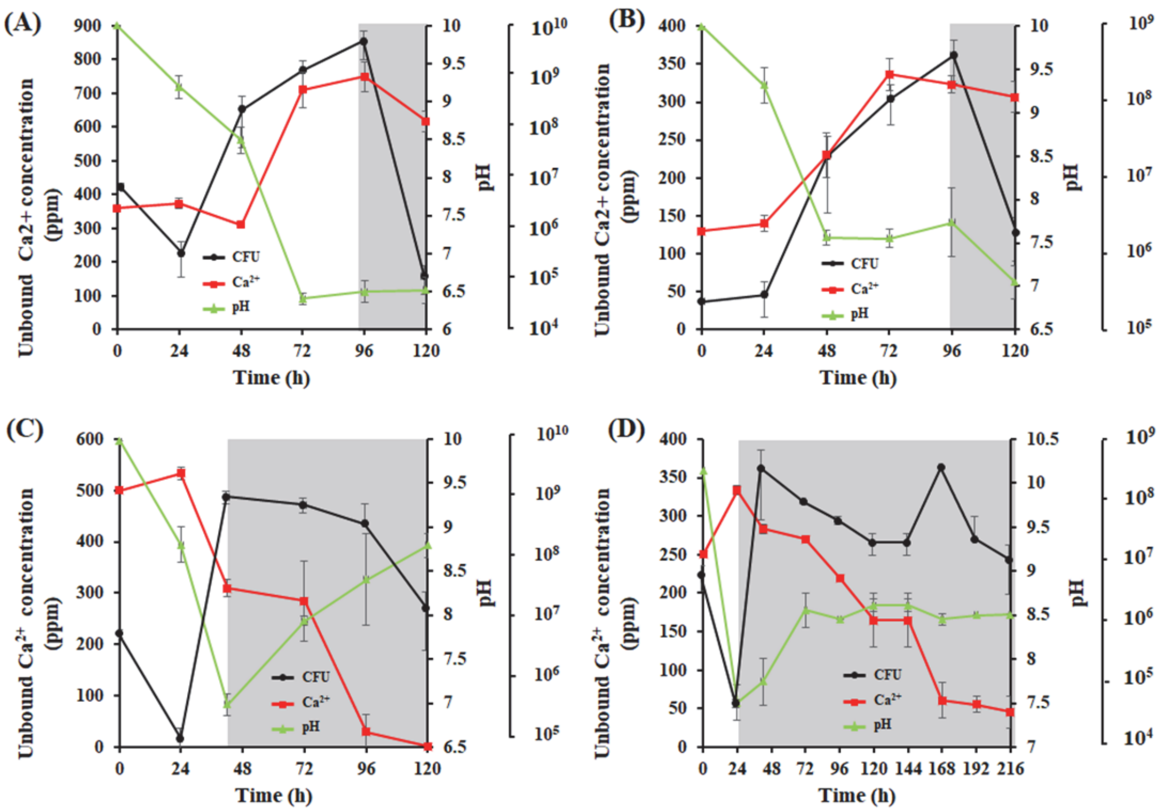

Fig. S1. Monitoring of growth, pH changes, and calcium utilization in B4 medium, B4L medium and modified B4, and B4L medium with pH adjustment to pH 10**. (A)** B4 medium. **(B)** B4L medium with calcium lactate. **(C)** Modified B4 medium with 0.2% glucose. **(D)** Modified B4L medium with 0.2% glucose.
